# Community-Scale Surveillance of SARS-CoV-2 and Influenza A Viruses in Wild Mammals, United States, 2022–2023

**DOI:** 10.3201/eid3108.241671

**Published:** 2025-08

**Authors:** Grete Wilson-Henjum, J. Jeffrey Root, Alex Worgo, Jeffrey Chandler, Robin Dyer, Jeff Flores, Jesse Morris, Ian Plummer, John Paul Seman, Kyle Van Why, Caleb Wellman, H. Bryant White, John Wilt, Diego Diel, Jon Heale, David L. Bergman, Josh Hewitt, Derek Collins, Ryan S. Miller, Steven Rekant, Kim M. Pepin

**Affiliations:** Utah State University S.J. and Jessie E. Quinney College of Natural Resources, Logan, Utah, USA (G. Wilson-Henjum, J. Hewitt); US Department of Agriculture Animal and Plant Health Inspection Service (APHIS) Wildlife Services, National Wildlife Research Center, Fort Collins, Colorado, USA (J.J. Root, A. Worgo, K.M. Pepin); US Department of Agriculture APHIS, Wildlife Disease Diagnostic Laboratory, Fort Collins (J. Chandler); US Department of Agriculture APHIS Wildlife Services, Augusta, Maine, USA (R. Dyer, J. Morris); US Department of Agriculture APHIS Wildlife Services, Sacramento, California, USA (J. Flores); US Department of Agriculture APHIS Wildlife Services, Portland, Oregon, USA (I. Plummer); US Department of Agriculture APHIS Wildlife Services, Sandusky, Ohio, USA (J.P. Seman, C. Wellman); Association of Fish and Wildlife Agencies, Phoenix, Arizona, USA (H.B. White); US Department of Agriculture APHIS Wildlife Services, Harrisburg, Pennsylvania, USA (K. Van Why, J. Wilt); Cornell University, Ithaca, New York, USA (D. Diel); US Department of Agriculture APHIS Wildlife Services, San Tan Valley, Arizona, USA (J. Heale); US Department of Agriculture APHIS Wildlife Services, Phoenix, Arizona, USA (D.L. Bergman); US Department of Agriculture APHIS Wildlife Services, National Wildlife Disease Program, Fort Collins (D. Collins); Centers for Epidemiology and Animal Health, Fort Collins (R.S. Miller); US Department of Agriculture Veterinary Services, Riverdale, Maryland, USA (S. Rekant)

**Keywords:** influenza, viruses, zoonoses, SARS-CoV-2, influenza A, respiratory infections, wildlife community, surveillance, detection, United States

## Abstract

Sampling of mammal communities across the United States during 2022–2023 detected evidence of SARS-CoV-2 antibodies in 3 new species and 2 previously described species and evidence of influenza A antibodies in 2 previously described species. Our analysis provides surveillance and sampling guidance for detection of rare exposure events.

Wildlife can transmit pathogens that threaten health of humans, domestic animals, and other wildlife (*1*). Wildlife disease surveillance can provide early warning of the changing epidemiology of rapidly evolving pathogens (*2*). In the United States, 2 rapidly evolving viruses with a broad host range have been detected in wildlife species: SARS-CoV-2 (*3*) and influenza A(H5N1) clade 2.3.4.4b (*4*). Coronaviruses and influenza A virus (IAV) both have a history of cross-species transmission and evolutionary events leading to strains that are highly virulent in multiple species and pandemic in humans (*5*,*6*).

Since January 2021, human-derived SARS-CoV-2 emerged and has been transmitting widely in wild cervids across North America (*7*) with evidence of spillback to humans (*8*). The virus has also emerged in domestic mink (*Neogale vison*) with transmission to sympatric free-roaming animals (*9*). Widespread distribution in animals and humans that are sympatric to wildlife species suggests risk for spillover and persistence in other wildlife. In addition, the host range of IAV has expanded to include marine mammals and seabirds (*10*) as well as cattle (*11*), which underscores the importance of understanding the changing host range of both SARS-CoV-2 and IAV in nature. We examined exposure to and co-infection of the 2 pathogens in wild mammal communities across different ecologic contexts.

## The Study

**Figure 1:**
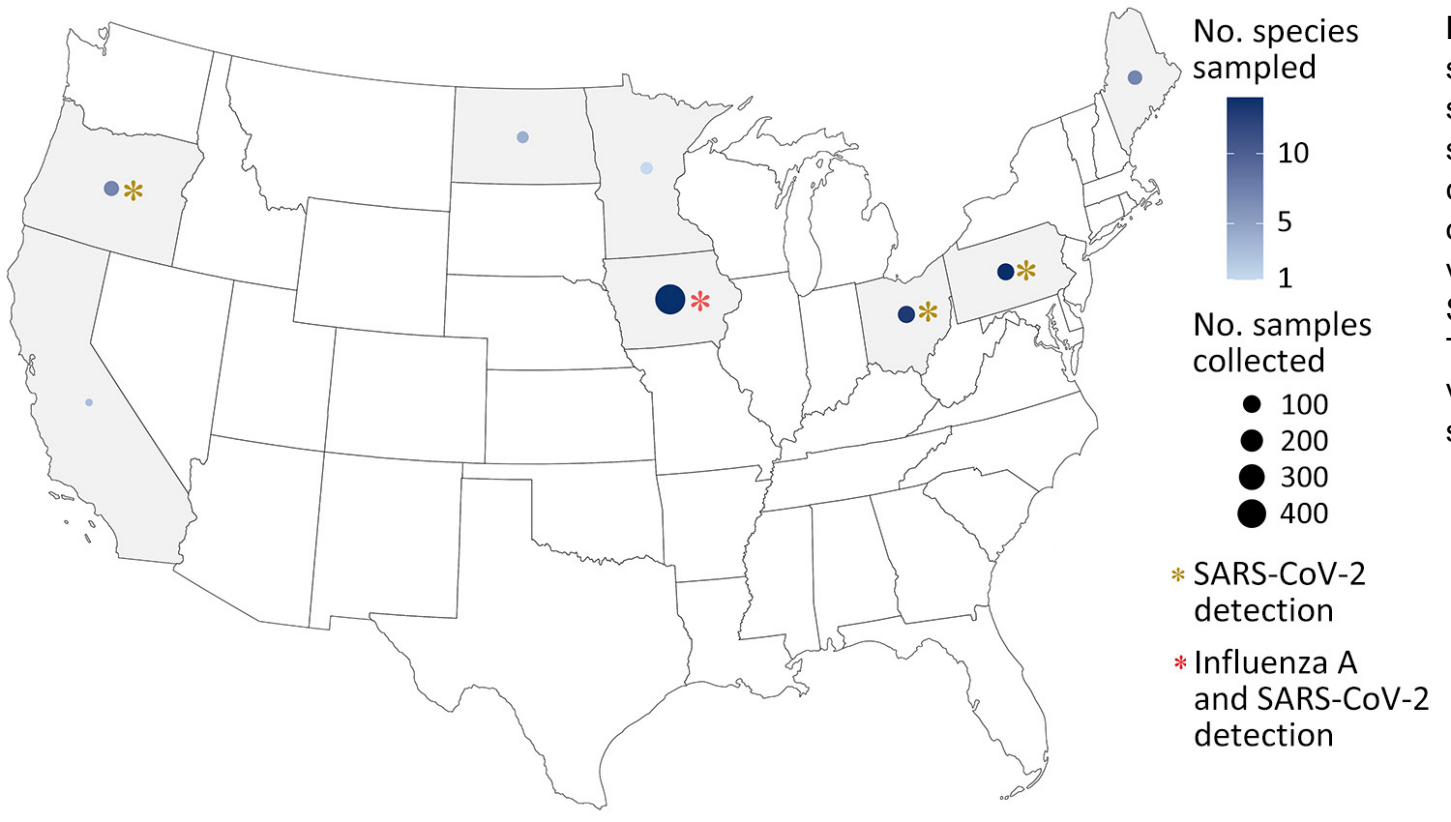
Locations of intensive sampling for SARS-CoV-2 samples collected across 28 species in 8 states during community-scale surveillance of SARS-CoV-2 and influenza A viruses in wild mammals, United States, October 2022–June 2023. The number of samples collected varied across each state and by species within each state.

**Figure 2:**
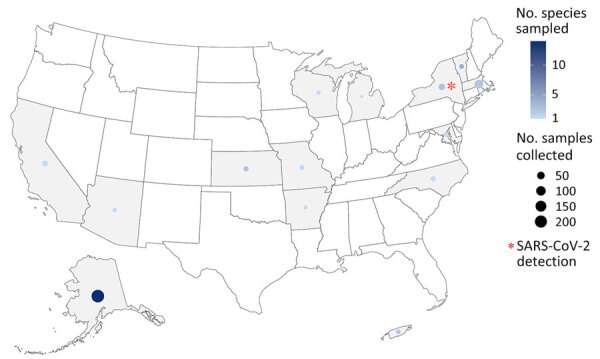
Locations of opportunistic sampling for SARS-CoV-2 samples collected across 17 species in 13 states and Puerto Rico during community-scale surveillance of SARS-CoV-2 and influenza A viruses in wild mammals, United States, September 2022–November 2023. The number of samples collected varied across each state.

We collected 1,172 samples from wildlife communities across the United States during September 2022–November 2023. Postmortem samples were collected opportunistically from 36 species in 20 states and Puerto Rico (Appendix 1 Table 1) by US Department of Agriculture Wildlife Services personnel during ongoing permitted management activity (Figure 1) and by the Association of Fish and Wildlife Agencies during Best Management Practices Trap Training (Figure 2); samples were taken from a variety of sympatric mammals in disparate locations. Where possible, we used continuous intensive sampling at the same location for >1 month to improve detection within a given mammal community.

Personnel collected swab samples and Nobuto strip blood samples from each animal (S. Bevins et al., unpub. data, https://doi.org/10.1101/2023.04.14.533542); we performed SARS-CoV-2 RNA preparation and subsequent detection using quantitative reverse transcription RT PCR (qRT-PCR) as previously described (*8*). US Department of Agriculture Animal and Plant Health Inspection Service National Veterinary Services Laboratories subjected nonnegative samples from novel hosts to confirmatory testing. We prepared SARS-CoV-2–specific neutralizing antibodies (NAbs) from Nobuto strips and detected using the Genscript cPass SARS-CoV-2 neutralization antibody detection kit (Thermo Fisher Scientific, https://www.thermofisher.com) as described (S. Bevins et al., unpub. data). We further investigated nonnegative samples from novel hosts by conventional viral neutralization testing (cVNT). We screened Nobuto eluates from intensively sampled sites (n = 747) (Figure 1) for IAV antibodies using a commercial blocking ELISA Influenza A MultiS-Screen Ab test (IDEXX Laboratories, https://www.IDEXX.com) (*12*) as described previously (*13*). We used the manufacturer’s recommended sample-to-negative ratio threshold of <0.5 to determine detection of IAV antibodies in serum (Appendix 2).

qRT-PCR testing detected SARS-CoV-2 RNA in 1 white-tailed deer (*Odocoileus virginianus*) sample (n = 45) and 2 nutria (*Myocastor coypus*) samples (n = 41) (Appendix 1 Table 2; Appendix 2 Figure 1). SARS-CoV-2 was not previously documented in nutria; because cycle threshold values were high, we used Sanger sequencing to verify the samples contained nutria host nucleic acid and were not contaminated by a sample from another species. After retesting, we did not have sufficient material for confirmatory testing. Additional sampling and testing are required to confirm nutria susceptibility to or SARS-CoV-2 presence in nutria populations.

**Figure 3:**
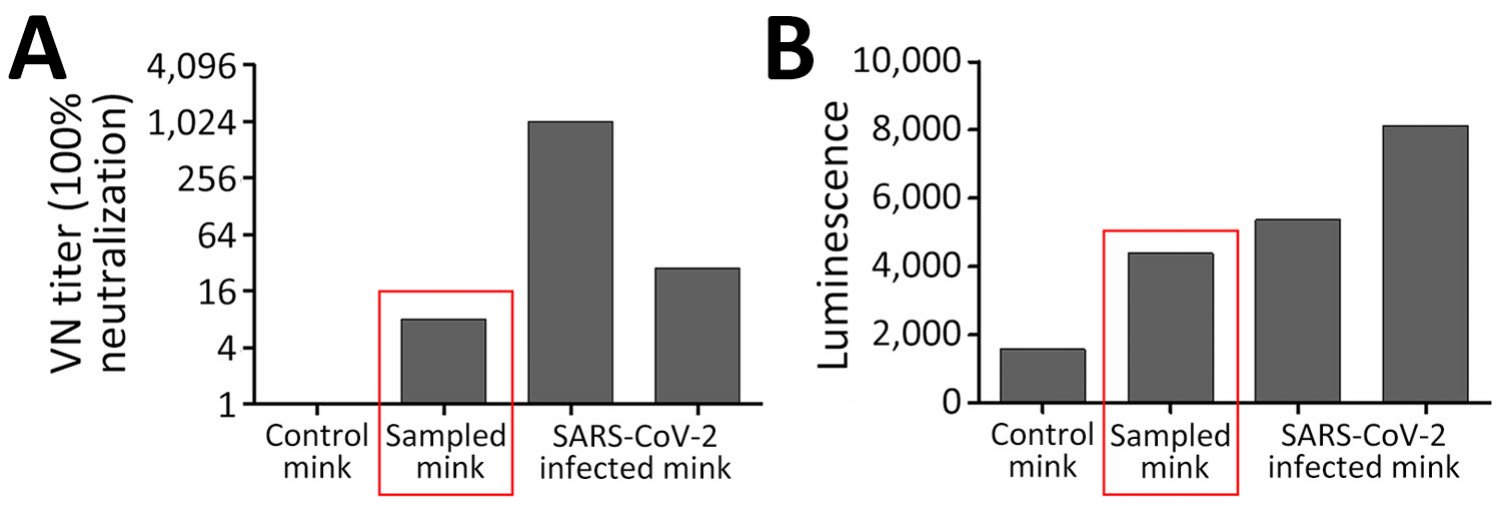
Results for testing conducted on a seropositive mink sample collected in Pennsylvania during community-scale surveillance of SARS-CoV-2 and influenza A viruses in wild mammals, United States, October 2022. A) Conventional VN testing; B) luciferase immunoprecipitation assay for nucleoprotein. The results from the 2 assays confirm previous exposure to the SARS-CoV-2 virus and rule out the likelihood that the immune response was generated in response to prior vaccination. Control mink data indicate results for a known negative-status mink, sampled mink data (red boxed) indicate results for our collected sample, and data for SARS-CoV-2–infected mink indicate results for known infected mink from samples collected outside of this study. VN, virus neutralization.

We found serologic evidence of SARS-CoV-2 exposure in 14 samples from 5 species (Appendix 1 Table 3): 1 coyote (*Canis latrans*; n = 25) (Appendix 2 Figure 2), 1 muskrat (*Ondatra zibethicus*; n = 41) (Appendix 2 Figure 1), 1 woodchuck (*Marmota monax*; n = 18) (Appendix 2 Figure 1), 1 domestic American mink (n = 13) (Appendix 2 Figure 1), and 10 white-tailed deer (n = 45) Appendix 2 Figure 1). Sample quality issues prevented the coyote Nobuto sample from cVNT testing. cVNT testing of other Nobuto eluates detected SARS-CoV-2 NAbs at dilution factors of 1:32 for muskrat and 1:8 for woodchuck (Appendix 1 Table 3). Because the mink sample was an escaped domestic mink from a farm that vaccinated its mink, we tested the sample for the Omicron BA.1 and B1 (variant D614G) strains of SARS-CoV-2. We detected NAbs for the B1 (variant D614G) strain at a dilution factor of 1:8 from the mink Nobuto eluate (Figure 3, panel A) but no response to Omicron BA.1 strain. Finally, N luciferase immunoprecipitation assay screening showed reactivity against the N protein (Figure 3, panel B; Appendix 1 Table 4), which indicates the animal was likely exposed to a pre-Omicron variant instead of or in addition to being vaccinated.

ELISA screening for IAV antibodies detected positive results in 7 raccoons (*Procyon lotor*; n = 270 across sites) (Appendix 2 Figure 3) and 1 Virginia opossum (*Didelphis virginiana*; n = 112 across sites) (Appendix 1 Table 5; Appendix 2). All positive animals were from the same site in Iowa within the Mississippi Flyway during 2 time periods (October 2022 and March 2023). Samples collected during October 2022 included 3 raccoon detections (n = 88; seroprevalence 3.4%) and the Virginia opossum detection (n = 40; seroprevalence 2.5%), whereas samples collected during March 2023 included 4 raccoon detections (n = 98; seroprevalence 4.1%). Previous opportunistic surveillance of avian IAVs in raccoons reported a similar seroprevalence in Maryland during 2004 (2.4%) but a higher seroprevalence in some western states: 25% in Wyoming during 2004 and 12.8% in Colorado during 2006 (*14*).

**Figure 4:**
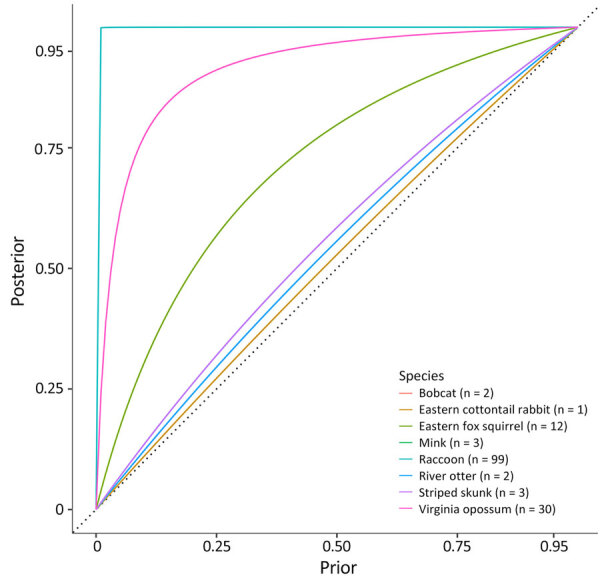
Posterior estimates of disease freedom varied based on prior input and the number of samples collected from each species in March 2023 in study of SARS-CoV-2 and influenza A viruses in wild mammals, United States. Collecting larger sample sizes enabled posterior estimates to depend less on prior inputs. The probability of disease freedom was analyzed for SARS-CoV-2–negative swabs from each species within each site. Probabilities for 8 species at a site in Iowa, United States, are shown.

In sites where no detections occurred, predictions of disease freedom were strongly influenced by the prior probability of disease freedom (i.e., site-level disease risk), but that influence was weakened by the sample size collected from each species within a site (Appendix 1 Table 6). We analyzed and illustrated the dependence between disease freedom estimates, sample size, and site-level disease risk at 1 site sampled in Iowa (Figure 4). Assumptions about site-level disease risk strongly determined disease freedom probability for species with <3 samples, such the eastern cottontail rabbit (*Sylvilagus floridanus*) with 1 sample. By comparison, disease freedom probability did not depend as greatly on site-level disease risk when >30 samples for a single species per site were collected, such as for raccoons or Virginia opossum.

## Conclusions

We did not find widespread SARS-CoV-2 occurrence in the wildlife communities, even for wildlife species sympatric with deer. We found evidence for infrequent incidence of SARS-CoV-2 exposure in novel species, highlighting the importance of appropriate site-level sample sizes for detection of rare exposure events. Our disease freedom analysis provides sampling guidance for detection of rare events in future surveillance programs. We did not find evidence of co-circulation of SARS-CoV-2 and IAVs in the same animals or species but did find sympatric exposure to IAVs in raccoons and Virginia opossum in the Mississippi Flyway. Community-scale wildlife disease surveillance is important for monitoring changing host ranges that can be realized given local ecologic contexts for rapidly evolving viruses and for refining risk-based surveillance designs.

Appendix 1 Data from community-scale surveillance of SARS-CoV-2 and influenza A viruses in wild mammals, United States.

Appendix 2 Additional information for community-scale surveillance of SARS-CoV-2 and influenza A viruses in wild mammals, United States.
