# Supplementary material for: Community-Scale Surveillance of SARS-CoV-2 and Influenza A Viruses in Wild Mammals, United States, 2022–2023
Source: Emerg Infect Dis. 2025 Aug;31(8):1625–9. doi: 10.3201/eid3108.241671 (PMC12309772; doi:10.3201/eid3108.241671)
Supplement: Appendix 2 — Additional information for community-scale surveillance of SARS-CoV-2 and influenza A viruses in wild mammals, United States. [file 24-1671-Techapp-s2.pdf]

Article DOI: https://doi.org/10.3201/eid3108.241671

EID cannot ensure accessibility for supplementary materials supplied by authors. Readers who have difficulty accessing supplementary content should contact the authors for assistance.

Community-Scale Surveillance of SARS-CoV-2 and Influenza A Viruses in Wild Mammals, United States, 2022–2023

Appendix

Sampling Methods

From September 2022 to November 2023, 1,172 samples were collected from 36 species across 20 states and Puerto Rico (Appendix 1 Table 1, https://wwwnc.cdc.gov/EID/article/31/8/24-1671-App1.xlsx). Four samples are from rats that were not identified to the species level and are designated as "Rat (Species Unknown)." Swab and Nobuto strip blood samples were collected from each animal as described (S. Bevins et al., unpub. data, https://doi.org/10.1101/2023.04.14.533542).

Samples were collected through two different surveillance designs: intensive sampling over time at a few sites, and opportunistic sampling over a broad spatial distribution without consistent site resampling, trading off sampling in time versus in space. The intensive sampling design collected samples alongside trapping at wildlife damage management sites and occurred multiple times per week over the course of a month or bi-weekly for 2 months (Appendix 2 Figure 1). Opportunistic samples were collected during trapper trainings across the U.S. (Appendix 2 Figure 2). All trapping and subsequent sampling took place during permitted wildlife management activities. Across both sampling protocols, additional opportunistic samples such as roadkill were also included for diagnostic testing. Nobuto strip samples collected from intensive sampling were subsequently screened for IAV (Appendix 2 Figure 3).

SARS-CoV-2 RNA preparation and subsequent detection using qRT-PCR was performed as previously described in Feng et al. (*1*). Nonnegative samples from novel hosts were subject to confirmatory testing at the USDA's National Veterinary Service Laboratory (NVSL). SARS-

CoV-2-specific neutralizing antibodies (NAbs) were prepared from Nobuto strips and detected using the Genscript cPass SARS-CoV-2 Neutralization Antibody Detection Kit (sVNT) as described in Bevins et al. Samples with percent inhibition exceeding 30 were considered positive for NAbs (*2*). Novel SARS-CoV-2 NAb detections by sVNT from the intensive sampling project were sent to NVSL for conventional virus neutralization testing (cVNT) (*3*). One Nobuto sample was collected from a domestic mink (*Neogale vison*) that escaped from a mink farm that had vaccinated the population. This sample, which tested positive by sVNT, was sent to the Animal Health Diagnostic Center at Cornell University for differentiating infected from vaccinated animals (DIVA) testing by cVNT testing to distinguish whether the positive was due to vaccination or viral infection. The cVNT assay uses SARS-CoV-2 variants representative of B1 (D614G), which is the target in the vaccine and would not have been circulating for a while before sample collection, and Omicron BA.1, which is more representative of circulating strains at the time of sample collection, and luciferase immunoprecipitation assay for N protein (N LIPS assay) screening (*4*).

Nobuto elutates from the intensively sampled sites (747 samples collected in 8 states from October 2022 through June 2023; Appendix 2 Figure 3) were screened for IAV antibodies via the IDEXX Influenza A MultiS-Screen Ab test, a commercial blocking enzyme-linked immunosorbent assay (bELISA) kit (*5*) at NWRC as described in Shriner et al. (*6*). This assay tests for presence of any IAV antibodies and does not distinguish a particular subtype (e.g., H5N1). A sample-to-negative (S/N) ratio threshold of <0.5 was used to determine detection of IAV antibodies during bELISA screening per the manufacturer's recommendation for poultry species. We were unable to optimize the S/N ratio threshold for the breadth of species sampled here due to our sampling design, however, bELISA tests are not considered species specific (*7*), and this particular kit is used to screen mammal species in field studies (*8*).

Analytical Methods

An analysis of disease freedom was conducted using the epiR package (https://cran.r-project.org/package=epiR) in program R version 4.2.2 (https://www.R-project.org) to assess the probability that disease was in fact not present in species and sites where SARS-CoV-2 RNA was not detected from swab samples. First the site-level sensitivity was calculated using sample

sizes and diagnostic test accuracy (sensitivity and specificity) for each species using methods described in Cameron et al. (*9*), implemented using the rsu.sep.rsfreecalc function in epiR. Disease freedom estimates also require site-level disease risk as an input parameter. Since our sampling represented the first SARS-CoV-2 surveillance event at each of the sites included, we had limited knowledge of previous disease circulation. The input design prevalence was therefore allowed to vary from 0.01 to 0.2 to account for gaps in information regarding previous levels of disease circulation. Species population sizes were approximated using an infinite population model, reflecting an assumption that sample sizes were small relative to the true, local population size. Test sensitivity and specificity were assumed to be 0.99 because data regarding these characteristics were unavailable. The range of output sensitivity values were fed into the rsu.pfree.rs function (*9*) to generate estimates of the probability of disease freedom. The lack of prior knowledge of possible disease introduction events were accounted for by allowing the prior probability of disease introduction to vary from 0 to 1, enabling assessment of the sensitivity of our sample size across a range of disease introduction scenarios. Inputs, sample sizes collected, and posterior estimates of disease freedom are listed in Appendix 1 Table 5.

References

1. Feng A, Bevins S, Chandler J, DeLiberto TJ, Ghai R, Lantz K, et al. Transmission of SARS-CoV-2 in free-ranging white-tailed deer in the United States. Nat Commun. 2023;14:4078. PubMed https://doi.org/10.1038/s41467-023-39782-x

2. Chandler JC, Bevins SN, Ellis JW, Linder TJ, Tell RM, Jenkins-Moore M, et al. SARS-CoV-2 exposure in wild white-tailed deer (*Odocoileus virginianus*). Proc Natl Acad Sci U S A. 2021;118:e2114828118. PubMed https://doi.org/10.1073/pnas.2114828118

3. Bewley KR, Coombes NS, Gagnon L, McInroy L, Baker N, Shaik I, et al. Quantification of SARS-CoV-2 neutralizing antibody by wild-type plaque reduction neutralization, microneutralization and pseudotyped virus neutralization assays. Nat Protoc. 2021;16:3114–40. PubMed https://doi.org/10.1038/s41596-021-00536-y

4. Burbelo PD, Riedo FX, Morishima C, Rawlings S, Smith D, Das S, et al. Sensitivity in detection of antibodies to nucleocapsid and spike proteins of severe acute respiratory syndrome coronavirus 2 in patients with coronavirus disease 2019. J Infect Dis. 2020;222:206–13. PubMed https://doi.org/10.1093/infdis/jiaa273

5. Brown JD, Stallknecht DE, Berghaus RD, Luttrell MP, Velek K, Kistler W, et al. Evaluation of a commercial blocking enzyme-linked immunosorbent assay to detect avian influenza virus antibodies in multiple experimentally infected avian species. Clin Vaccine Immunol. 2009;16:824–9. PubMed https://doi.org/10.1128/CVI.00084-09

6. Shriner SA, VanDalen KK, Root JJ, Sullivan HJ. Evaluation and optimization of a commercial blocking ELISA for detecting antibodies to influenza A virus for research and surveillance of mallards. J Virol Methods. 2016;228:130–4. PubMed https://doi.org/10.1016/j.jviromet.2015.11.021

7. Vandalen KK, Shriner SA, Sullivan HJ, Root JJ, Franklin AB. Monitoring exposure to avian influenza viruses in wild mammals. Mammal Rev. 2009;39:167–77. PubMed https://doi.org/10.1111/j.1365-2907.2009.00144.x

8. Ramey AM, Beckmen KB, Saalfeld DT, Nicholson KL, Mangipane BA, Scott LC, et al. Exposure of Wild Mammals to Influenza A(H5N1) Virus, Alaska, USA, 2020-2023. Emerg Infect Dis. 2025;31:804–8. PubMed https://doi.org/10.3201/eid3104.241002

9. Cameron AR, Baldock FC. A new probability formula for surveys to substantiate freedom from disease. Prev Vet Med. 1998;34:1–17. PubMed https://doi.org/10.1016/S0167-5877(97)00081-0

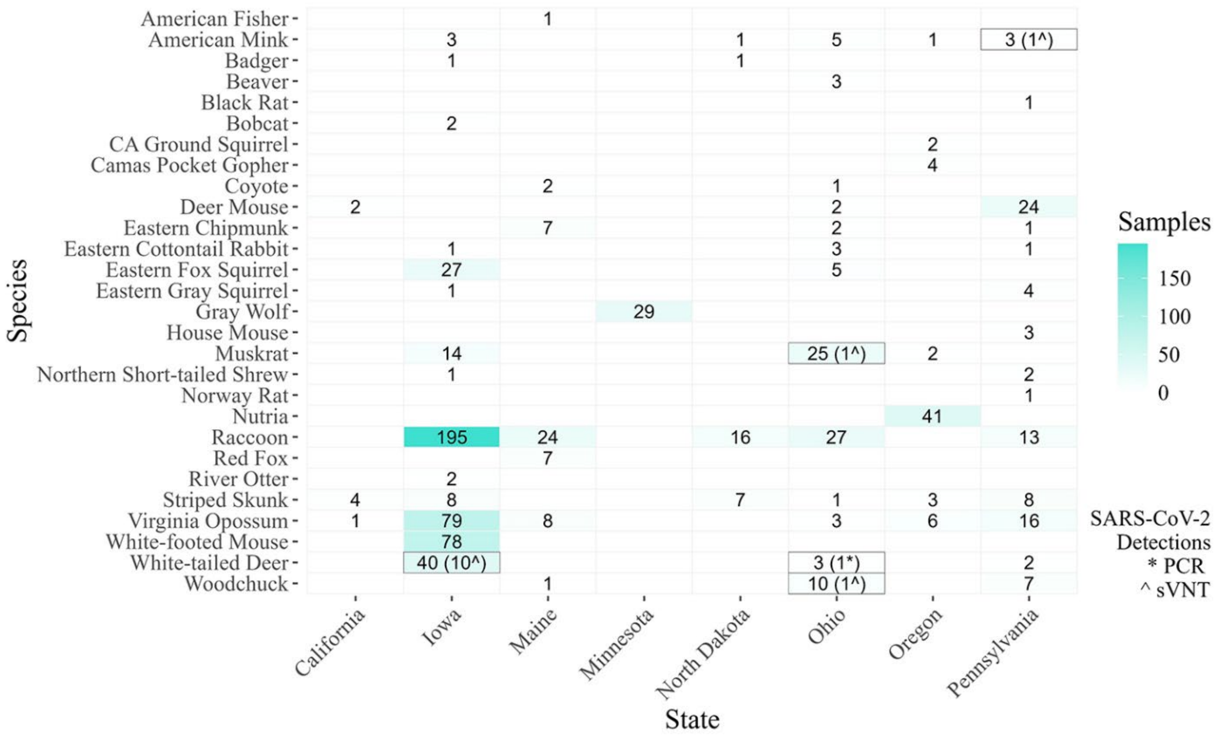

Appendix 2 Figure 1. Data from community-scale surveillance of SARS-CoV-2 and influenza A viruses in wild mammals, September 2022–November 2023. The number of samples from each species within each state are shown. The number of nonnegative SARS-CoV-2 detections for each species within each state is reported in parentheses. sVNT, surrogate virus neutralization test.

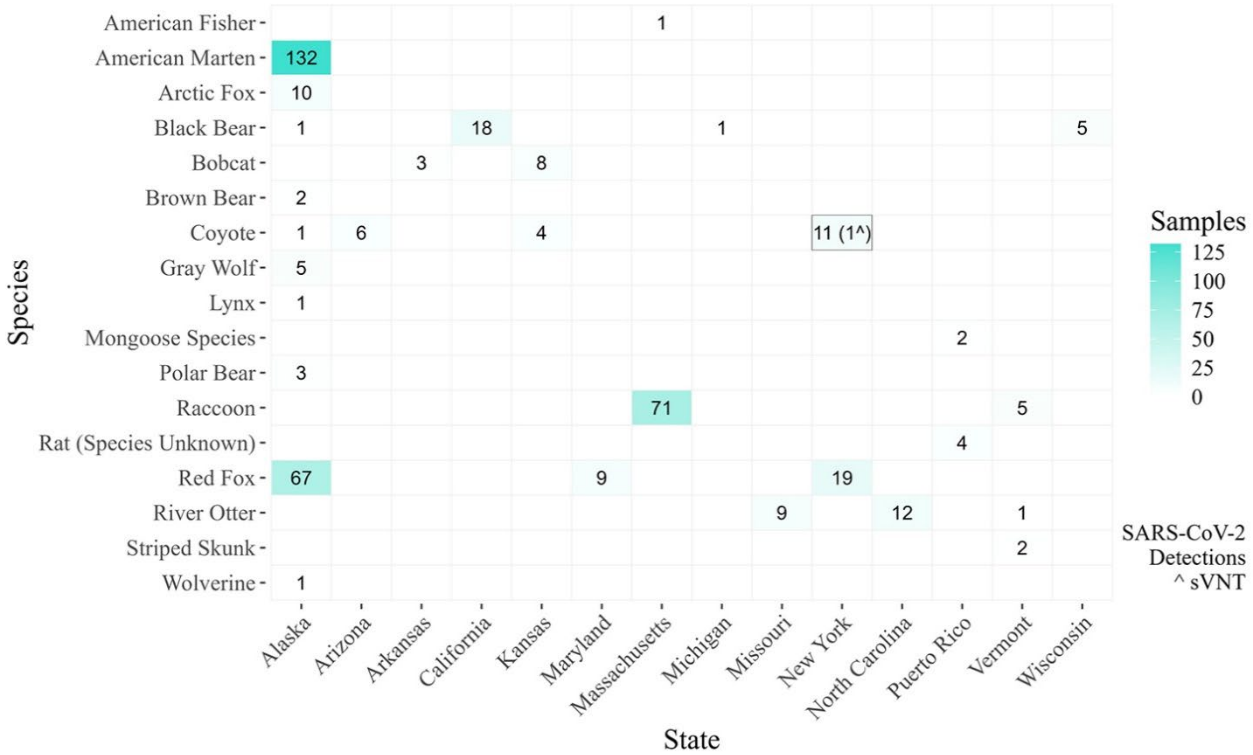

Appendix 2 Figure 2. Data from community-scale surveillance of SARS-CoV-2 and influenza A viruses in wild mammals, September 2022–November 2023. The number of samples from each species within each state are shown. The number of nonnegative serodetections for each species within each state is reported in parentheses. sVNT, surrogate virus neutralization test.

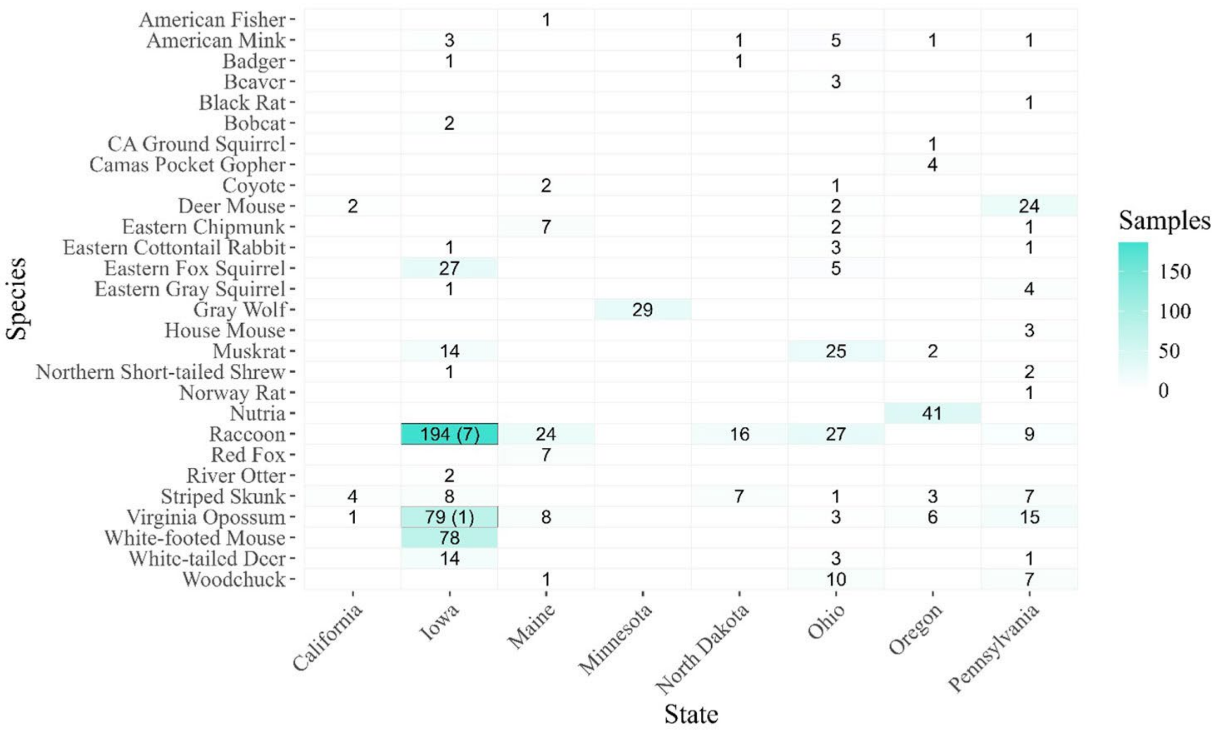

Appendix 2 Figure 3. Data from community-scale surveillance of SARS-CoV-2 and influenza A viruses in wild mammals. Intensive sampling was conducted collected across 28 species in 8 states during October 2022–June 2023. The number of samples collected varied across each state and species. Samples were screened for Influenza A antibodies via the IDEXX Influenza A MultiS-Screen Ab test, a commercial blocking ELISA kit. The number of nonnegative IAV ELISA detections per each species within each state is reported in parentheses.
